# Update of cellular responses to the efferocytosis of necroptosis and pyroptosis

**DOI:** 10.1186/s13008-023-00087-6

**Published:** 2023-04-09

**Authors:** Chandra Agung Purnama, Anna Meiliana, Melisa Intan Barliana, Keri Lestari

**Affiliations:** 1 Department of Pharmacology and Clinical Pharmacy, Faculty of Pharmacy, Universitas Padjadjaran, Jl. Ir. Soekarno Km 21, Jatinangor, 45363 Indonesia; 2 Prodia Clinical Laboratory, Jl. Supratman No. 43, Bandung, 40114 Indonesia; 3 Prodia Education and Research Institute, Jl. Kramat Raya No 150, Jakarta, Indonesia; 4 Department of Biological Pharmacy, Faculty of Pharmacy, Universitas Padjadjaran, Jl. Ir. Soekarno Km 21, Jatinangor, 45363 Indonesia; 5 Centre of Excellence for Pharmaceutical Care Innovation, Universitas Padjadjaran, Jl. Ir. Soekarno Km 21, Jatinangor, 45363 Indonesia

**Keywords:** Efferocytosis, Necroptosis, Pyroptosis, Cell death, Inflammation

## Abstract

Cell death is a basic physiological process that occurs in all living organisms. A few key players in these mechanisms, as well as various forms of cell death programming, have been identified. Apoptotic cell phagocytosis, also known as apoptotic cell clearance, is a well-established process regulated by a number of molecular components, including ‘find-me’, ‘eat-me’ and engulfment signals. Efferocytosis, or the rapid phagocytic clearance of cell death, is a critical mechanism for tissue homeostasis. Despite having similar mechanism to phagocytic clearance of infections, efferocytosis differs from phagocytosis in that it induces a tissue-healing response and is immunologically inert. However, as field of cell death has rapid expanded, much attention has recently been drawn to the efferocytosis of additional necrotic-like cell types, such as necroptosis and pyroptosis. Unlike apoptosis, this method of cell suicide allows the release of immunogenic cellular material and causes inflammation. Regardless of the cause of cell death, the clearance of dead cells is a necessary function to avoid uncontrolled synthesis of pro-inflammatory molecules and inflammatory disorder. We compare and contrast apoptosis, necroptosis and pyroptosis, as well as the various molecular mechanisms of efferocytosis in each type of cell death, and investigate how these may have functional effects on different intracellular organelles and signalling networks. Understanding how efferocytic cells react to necroptotic and pyroptotic cell uptake can help us understand how to modulate these cell death processes for therapeutic purposes.

## Background

Every day, approximately 37.2 trillion cells die in the adult body, the majority which are cause by the caspase-dependent apoptosis mechanism [1]. This apoptotic mechanism has been determined to be essential for the protection of cellular functions in physiological, embryogenesis, tissue repair and also the restoration of homeostasis after disease and inflammation have subsided in a variety of tissues [1, 2]. As an example, neutrophils, the primary innate immune cells that serve as the body’s primary defence mechanism against pathogen attack, have a relatively short lifespan (24 h) and a daily turnover rate of more than 100 billion. Millions of immature T lymphocytes or B lymphocytes are synthesised in the thymus or bone marrow, but only a small percentage mature; the rest are destroyed by apoptosis. Apoptosis aids in the reorganisation of tissues during organs and embryo development. Furthermore, as part of our daily physical cleaning, many ‘used’ cells, such as ageing red blood cells, are discarded [3]. Apoptosis occurs after neutrophils, monocytes and lymphocytes that infiltrate inflammatory disorders have completed their function. As the primary pathogen, serious COVID-19 affects the process of apoptosis in virus-infected cells. The influx of monocytes, macrophages and T lymphocytes into the lungs defined severe COVID-19 [1, 3]. Pathogen infection can cause pathogen cells to die, triggering phagocytic removal in either an immunologically silent or pro-inflammatory mode [4].

Cell death is classified into two types: regulated (apoptosis) and non-regulated (necrosis). Necrosis is the medical term for the unintentional death of cells caused by significant physiological or chemical trauma, such as membrane shearing and breakage caused by high temperatures, osmotic pressure, acidity, or contact with substances such as surfactants and endotoxins. Necrotic cell death, can be either accidental or programmed (for example, pyroptosis and necroptosis) [5]. Programmed necrosis is a type of genetically regulated cell death characterised by morphological characteristics such as cellular enlargement (oncosis), membrane rupture and release of cellular content release. According to new research, certain signalling pathways are required to mediate the programmed necrosis carried out by various death signals, such as necroptosis triggered by the tumor necrosis factor (TNF) superfamily cytokines, interferon and T-cell receptors, toll-like receptors (TLRs), cellular metabolic and genotoxic stresses. Terms like ‘programmed necrosis’ have been employed to highlight the fact that necrotic cell injury is a ‘programmed’ form of cellular death rather than merely an ‘accidental death’. Specific substances (such as death cytokines and their related death receptors) and a sophisticated biochemical signalling cascade are among the triggers that cause necrotic cell injury [6]. Necrosis is distinct from apoptosis because necrotic cells do not transform into apoptotic bodies, which requires enzyme activity. Importantly, these differences suggest that the methods for removing cell debris produced by necrosis versus apoptosis may differ significantly [5].

**Fig. 1:**
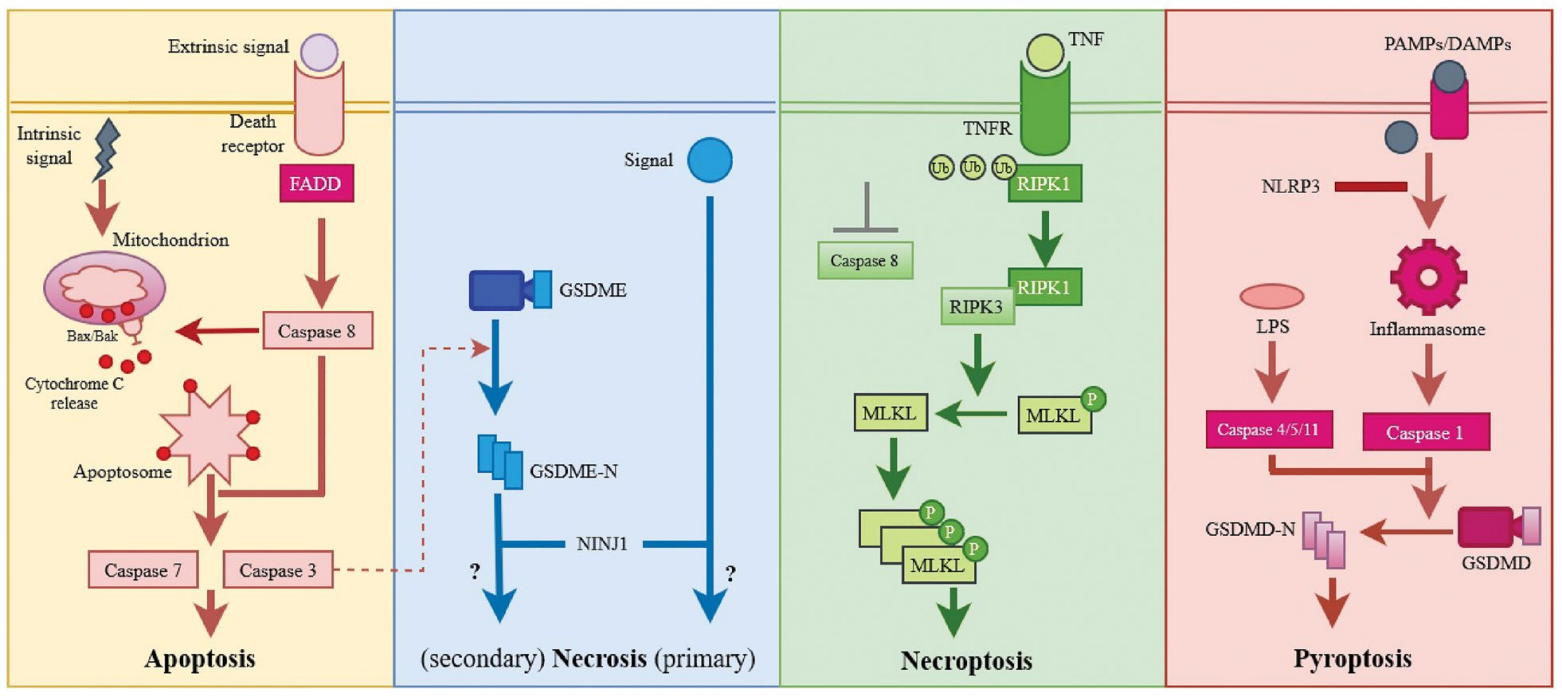
Cell death molecular mechanisms schematic. Both intrinsic and extrinsic apoptotic signals activate death receptors on the cell surface, which activates caspase 3/7. The progression of secondary necrosis may be accelerated by caspase-3-cleaved GSDME and NINJ1. Primary necrosis typically has a randomness, resulting in an uncontrolled rupture of membranes. Through its interaction with the INF-R, TNF-may induce necroptosis by activating RIPK1/3 and creating MLKL membrane holes. Pyroptosis is triggered by the detection of PAMPs/DAMPs like ATP. Pyroptosis is induced by caspase 1, and caspases 4/5/11 converge with the activation of Gasdermin D

Apoptosis was the first recognised form of programmed cell death, and it is frequently immune-silent due to apoptotic effectors such as caspase 3 and 7 [7]. When cell death receptors are activated, extrinsic apoptosis occurs, whereas intrinsic apoptosis occurs when cellular stress activates the mitochondrial pathway. Pro-caspase 8 is drawn to the intracellular signalling complex during apoptosis by the adaptor protein FADD (FAS associated via death domain). Caspase 9 is activated and cytochrome c is released via the intrinsic route. Caspases 3 and 7 activation results in the development of both apoptotic pathways. Find-me signals are released when caspases are activated during apoptosis to guide apoptotic cells toward phagocytes [8]. This procedure is depicted in Fig. 1. The lytic forms of cell death necroptosis and pyroptosis, on the other hand, allow the secretion of immunostimulatory substances. According to genetic evidence, these cell death mechanisms have the potential to cause severe inflammatory reactions in living organisms, contributing to the pathology of a variety of inflammatory diseases. Necroptosis and pyroptosis are pro-inflammatory pathways because they promote excessive escape of cell contents, including damage-associated molecular patterns (DAMPs) [9]. Necroptosis is distinguished from other types of necrosis by the presence of receptor-interacting protein kinase 1 (RIPK1) and RIPK3, which attract and phosphorylate the mixed lineage kinase domain-like protein (MLKL). MLKL then oligomerises and moves to the inner leaflet of the plasma membrane, where it stimulates membrane permeabilization and cell death [5]. Excessive inflammasome activation, on the other hand, causes pyroptosis. Inflammasome-expressing cell types, such as macrophages, are exposed to it following infection (with the intracellular pathogen *Salmonella typhimurium*) or LPS treatment [10]. Inflammasomes containing activated caspases 1 or 11 form gasdermin D pores by processing the precursor of gasdermin D pores at the plasma membrane. These pores allow IL-1 to be released, but they also permeate the plasma membrane excessively, causing cell lysis and pyroptosis [5]. Nonetheless, necroptotic and pyroptotic cells must be eliminated quickly and without causing inflammation.

Despite the fact that the vast majority of apoptotic cells are produced on a regular basis, they are rarely seen in tissues in vivo. This is because of the fascinating relationship between cell death and efferocytosis, the phagocytic process that effectively removes apoptotic cells [2]. Professional and non-professional phagocytic cells use efferocytosis to remove apoptotic cells quickly and effectively [11]. Due to efferocytosis, early multicellular creatures were able to control their growth by eliminating dead cells as they developed. Efferocytosis is required for development and growth, as well as to reduce inflammation and maintain cellular homeostasis [12, 13]. The mechanisms of efferocytosis are distinct from those of standard phagocytosis, both visually and mechanistically. Phagocytosis necessitates the expression of signals and receptors on the phagocyte, cytoskeleton reorganisation to ingest apoptotic cells and induction of phagosome-lysosome fusion to destroy the content of apoptotic cells [8]. When the number of apoptotic cells exceeds the number of accessible classical phagocytes, as occurs during acute inflammatory responses, phagocytes must be able to consume them quickly, a process known as constant efferocytosis. In this case, macrophages regulate inflammation to aid tissue recovery. When efferocytosis is inadequate, this process is ineffective and causes tissue injury [14].

The majority of the contents of an infected apoptotic cell (such as proteins, nucleic acids and lipids) are similar to those of a non-infected dead cell; however, efferocytes such as macrophages can detect the difference, allowing the efferocyte to produce an effective immune response against pathogens within efferocytes cells [13]. This demonstrates how efferocytosis differs from phagocytosis and how efferocytes can tell the difference between harmful and apoptotic cargo. Defective efferocytosis can lend to the accumulation of apoptotic cells in inflammatory foci, resulting in necrosis, cytolysis and the production of intracellular contents tissue damage. Lesional efferocytosis and larger necrotic cores were associated with low-density lipoprotein receptor deficiencies in chronic diseases when compared to healthy animals. Inadequate efferocytosis is a common diabetic side effect that can prevent tissue repair and lead to chronic inflammation as a result of a build-up of apoptotic cells at the wound site [15]. The molecular pathway of efferocytosis in necroptosis and pyroptosis is less well understood than the apoptosis mechanism. We discuss what is known about the efferocytosis process in necroptosis conditions such as inflammatory diseases and pathogen infection-induced pyroptosis, as well as the differences between these and apoptosis and their effects on organism physiology. This mechanism ‘find-me’ and ‘eat-me’, signals, as well as engulfing dead cells.

## Hallmark of infection and inflammation

Understanding the distinctions between infection and inflammation mechanism is the first step towards understanding necroptosis and pyroptosis mechanisms. Inflammation occurs as a complex biological response when vascular tissues come into contact with pathogens, dying cells, or irritants. Some of the most common symptoms acute inflammation include swelling (tumour), heat (calor), pain (dolour), redness (rubor) and loss of function (functio laesa). Inflammation is the body’s reaction to potentially harmful stimuli in an attempt to rid itself of them and begin the healing process. Even when caused by an infection, inflammation is not always synonymous with infection. Even though a bacterium causes an infection, inflammation is one of the pathogen’s responses. Inflammation, on the other hand, is a stereotypical reaction, so it is classifies as innate immunity rather than adaptive immunity, which is tailored to each specific pathogen [16]. In the same way, an infection is defined as the invasion of tissues by pathogenic organisms, their proliferation and the host tissues’ response to their toxins. Infections are caused by bacteria, viruses, prions and viroid’s, as well as larger organisms such as parasites and fungi. The immune system in hosts enables pathogen defence. Mammalian hosts respond to infections by first undergoing an innate response, which frequently includes inflammation, and then an adaptive response. As a result, while inflammation is not always associated with infection, infection is usually associated with inflammation [17].

The host detects the presence of microorganisms through a variety of mechanisms, the most important of which include various receptor families. These numerous sensors are constantly scanning for microorganisms in various subcellular compartments. Several Toll-like receptors (TLR) and C-type lectins are found in the plasma membrane and can detect the presence of bacteria in extracellular fluids [18, 19]. Each of the 11 distinct TLRs recognises microbial components that are chemically distinct from those produced by the host (also known as PAMPs—pathogen-associated molecular patterns). TLR4, recognises lipopolysaccharides, which are membrane components found in the majority of gram-negative bacteria. TLR2 recognises lipopeptides, which are found in many gram-positive organisms. By focusing on these conserved pathogen-associated molecular patterns, the host is able to accomplish two remarkable and significant feats. Despite the enormous diversity of these organisms, it can distinguish between self and nonself components by employing a relatively small number of receptors capable of identify the majority of bacteria, fungi and viruses [18].

The inflammatory response clearly contributes to an infection. Vasodilatation greatly accelerates the delivery of blood-borne defences to the affected site. Increased vascular permeability allows soluble immune proteins such as complement and antibody to enter the environment and fight invasive microorganisms. Neoexpressed vascular adhesion molecules and chemokines attracts leukocytes into the tissue space. When pathogens arrive at the infection site, leukocytes digest them and try to get rid of them. As a result, both soluble and cellular immune responses are rapidly provided by these systems [20].

The immune system has evolved several mechanisms to detect cell death and warn of potential dangers. An infection, such as a toxin or a cytopathic bacterium, can cause necrotic cell death. Even if it is not directly caused by a pathogen, cell death frequently occurs in a location where microbes have been introduced as a result of an injury, such as a penetrating wound. Because of their 20-min doubling time, microbes pose a massive threat that requires an immediate response to contain. When the host detects dead cells, it immediately initiates an inflammatory response to neutralise the threat [20]. However, in a variety of other circumstances, cells die and cause inflammation in the absence of a microbial infection. When a tumour is forming, for example, dying cells may still be harmful to the body and may be eliminated by the host immune system [21]. Other times, the inflammatory response may work against some disease processes in different ways. Apoptosis can occasionally be quiet or inflammatory in chronic situations, such as diabetes. It is thought that how quickly phagocytes remove apoptotic cells is an important factor. If apoptotic cells are not given enough time to clear, they undergo secondary necrosis, allowing macromolecules to pass through their membranes. If this process occurs before the phagocytes digest the dead cells, the pro-inflammatory intracellular contents of the dead cells will be released, triggering a host response [20]. Because harmful activities, such as infections, can cause cells to die, this inflammatory response to apoptotic cells may be beneficial teleologically.

## Death cell in necroptosis and pyroptosis

### Necroptosis

Necroptosis can be aided extrinsic apoptotic receptors. When apoptosis is inhibited, a process known as necroptosis takes place, which results in cellular self-destruction. Necroptosis differs from other types of programmed necrotic cell death in that it occurs without caspase activation. Necroptosis is a non-apoptotic, the core necroptotic pathway is triggered by the proteins RIPK3 and MLKL (Fig. 1). Necroptosis is thought to be a type of controlled necrosis and is considered inflammatory because to lacks the find-me and eat-me signals of apoptosis. The RIPK1 recruits and phosphorylates RIPK3, forming the RIPK1/RIPK3 complex, and it activates MLKL and releases DAMPs for phagocytes to recognise [22, 23]. Multiple sclerosis and amyotrophic lateral sclerosis, both of which cause cell death and inflammation, have recently been linked to necroptosis in humans [24–27].

Both apoptosis and necroptosis are regulated by the same molecular machinery [28]. Apoptosis is a natural biological process that allows the majority of cells in the body to keep their internal environment stable by preserving a specific developmental pathway. As a result, apoptosis is a tightly regulated process of irreversible caspase-dependent cell death, whereas necroptosis is caspase-independent signalling pathway that is primarily dependent on the RIPK1/RIPK3/MLKL complex [29]. Apoptosis is characterised by cell shrinkage, membrane blebbing, chromatin condensation, the formation of apoptotic bodies and rapid phagocytosis by nearby phagocytes. Because there is no materials overflow during apoptosis, there is no inflammatory immunological reaction. Necroptosis, on the other hand, is a type of cytolytic death. As a result of the plasma membrane’s rapid loss of integrity, pro-inflammatory molecules escape from the cells, triggering a variety of inflammatory reactions [28–30]. Furthermore, all necroptosis pathways share one feature: they all prevented by caspase 8 activation (Fig. 1).

Necroptosis occurs frequently when components of the death receptor (DR) apoptotic signalling pathway fail. For example, in order to trigger DR Necroptosis, the majority of studies to date have suppressed cIAP1/2 and caspase 8, two cellular proteins that generally ubiquitylate or cleave RIPK1/3 to prevent necroptosis [31]. When caspase-8 and/or cIAPs are not expressed, activation of RIPK1/3 by DRs (such as TNFR1, TRAIL-R, CD95), Toll-like receptors (such as TLR3 or TLR4), or the cytosolic Z-DNA/Z-RNA detecting receptor, Z-DNA binding protein 1 (ZBP1/DAI/DLM-1), can instantly cause necroptosis. Activation of these receptors leads to the establishment of a RIPK1-RIPK3 cell death platform known as the necrosome by interacting with heteroamyloid-structured RIPK1/RIPK3 RHIM-RHIM domains [32]. The necroptotic executioner MLKL is phosphorylated by RIPK3, allowing MLKL to oligomerise and associate with membranes, potentially damaging the plasma membrane and releasing DAMPs [33–35]. In many cases, RIPK1 and its kinase activity are critical for necroptosis; however, recent research has shown that the RHIM, a RIPK1 scaffolding role, functions as a critical blocker of fatal necroptotic death by inhibiting ZBP1 binding and RIPK3 oligomerisation [36, 37]. Non-necroptotic transcriptional (such as activating inflammatory cytokines) and post-translational (such as activating apoptotic cell death) activities of RIPK1 and RIPK3 should also be considered [38]. As a result, the requirement for MLKL is the best way to describe mammalian necroptosis.

### Pyroptosis

Pyroptosis is made up of the words ‘pyro’ and ‘ptosis’. Pyro means ‘fire’, which refers to the inflammatory properties of pyroptosis, and ‘ptosis’ means ‘falling’, which is related to other types of programmed cell death. Apoptosis and pyroptosis share two characteristics: DNA damage and chromatin condensation [39]. Interestingly, pyroptotic cells had multiple bubble-like protuberances on the exterior of the cell membrane’s exterior that were inflated until they ruptured [40]. Apoptosis, like membrane blabbing, results in this process and caspase 3 is required for it [41–44]. Specific morphological characteristics distinguish pyroptosis from apoptosis. Although the pyroptotic cells undergo chromatin condensation and DNA fragmentation, their nucleuses remain intact. The integrity of the nucleus and a bit of DNA laddering are present during pyroptosis. The inflammation-induced pore formation results in swelling and osmotic lysis in pyroptotic cells. In comparison to pyroptotic cells, cells undergoing apoptosis maintain intact membranes. Although pyroptosis can cause inflammation when triggered by external or intracellular triggers such as bacteria, viruses, toxins and chemotherapeutic drugs, apoptosis is widely accepted to be a benign type of cell death [45]. In fact, pyroptosis, as opposed to necrosis, allows the cytoplasm to flatten as a result of plasma membrane leakage [40]. In contrast to necroptosis, pyroptosis is induced response to infection, such as caspases like caspases 1, 4 and 5 detect lipopolysaccharide on intracellular gram-negative bacteria (Fig. 1). When activated, this caspase cleaves gasdermin D (GSDMD), resulting in cell lysis. Pyroptosis and necrosis cause the released as a result of pro-inflammatory cytokines [30].

Caspase activation or granzyme release results in the N-terminal oligomerisation of gasdermin and the formation of a pore (1–2 m diameter) in the plasma membrane, allowing mature IL-1/IL-18 with a diameter of 4.5 nm and caspase-1 with a diameter of 7.5 nm, respectively [46]. Water leaking through the perforations causes cell swelling and osmotic lysis, rupturing the plasma membrane and releasing IL-1 and IL-18. The pyroptotic cells are permeable to several dyes due to their low molecular weight, including 7-aminoactinomycin (7-AAD), ethidium bromide and propidium iodide [47]. Apoptotic cells, unlike pyroptotic cells, maintain the integrity of their membranes, preventing these dyes from staining them [48–50]. Annexin V stains both apoptotic and pyroptotic cells, and the colour binds to phosphatidyl serine (PS). As a result, Annexin V cannot tell the difference between apoptotic and pyroptotic cells. Furthermore, pyroptosis causes the formation of pyroptotic bodies, whereas apoptosis causes the formation of apoptotic bodies. It is worth noting that pyroptotic entities have a 1–5 m diameter, which is comparable to the size of apoptotic bodies [51].

Numerous caspases, including caspase 11 and its human orthologs caspase 4 and 5, as well as the apoptotic effector caspase 3 [52, 53], have been shown in studies to be capable of inducing pyroptosis, also known as caspase-1-mediated cell death. The ability of these caspases to cleave and stimulate specific components of the pore-forming gasdermin gene family, which includes six genes in humans and ten genes in rats, mediates pyroptotic cell death. Caspases-1/4/5/11 have been found to target GSDMD, whereas caspase-3 can handle GSDME/DFNA5 (Fig. 1) [54, 55]. The cleavage of the linker domain of gasdermin between the N- and C-termini separates an active N-terminal area from inhibitory C-terminal fragment. Thus, expression of the gasdermin-N domain from GSDMA, GSDMA3, GSDMB, GSDMAC, GSDMD and GSDME alone could indicate cell death; however, more research into the physiological functions of these gasdermins, as well as possible proteolytic enzymes that target GSDMA to GSDMC, is needed [46, 54]. However, when the gasdermin-N domain is released by proteolysis, it interacts with acidic phospholipids, such as phosphoinositides found on the inner leaflet of the mammalian plasma membrane, and forms oligomeric death-inducing holes [46, 56–58]. While in vivo studies show that bacteria can survive pyroptosis and are removed by neutrophils, in vitro studies show that gasdermins can target bacterial membranes to induce lysis. [59, 60] Regardless, we can now say that gasdermin is required for mammalian cell pyroptosis.

Inflammasomes, which are intracellular multiprotein signalling complexes, are activated when inflammatory ligands are detected, according to the classical theory of caspase-1 mediated pyroptosis (Fig. 1). AIM2 (absent in Melanoma 2), Pyrin and the NOD-like receptor family members NLRP1, NLRP3 and NLRC4 are among the most extensively studied inflammasome sensors [61]. For example, cytosolic double-stranded DNA binds to and activates the AIM2 inflammasome. The NLRP3 inflammasome reacts with a wide range of molecules, including ATP, crystalline substances (such as cholesterol crystals) and viral elements, which when combined cause potassium efflux and the subsequent association of NEK7 (NIMA-related kinase 7) with NLRP3 to cause NLRP3 activity [62, 63]. Caspase-1 is frequently attracted to inflammasome sensor like NLRP3 via the adaptor protein ASC, which contains a CARD-domain. As a result of this recruitment, the inactive caspase-1 species, p46 and p33/p10 subunits, are automatically processed into their catalytically active forms [64].

**Fig. 2:**
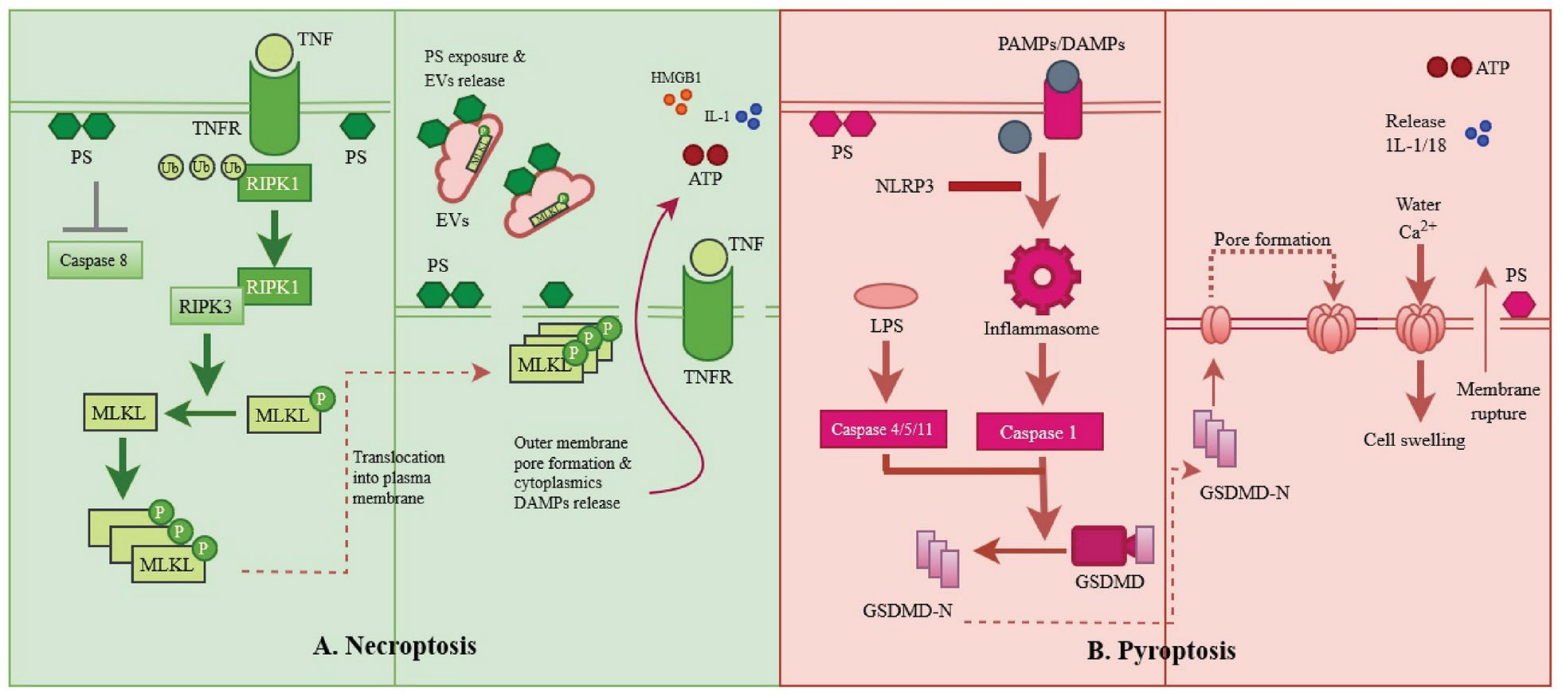
Mechanism of find-me and eat-me signal release in necroptosis and pyroptosis. **A** Immunomodulatory signal is induced by MLKL phosphorylation. PS is exposed to the outer membrane, and extracellular vesicles are released together with necroptotic bodies as a result of the creation of MLKL pores, which also causes the release of cytoplasmic DAMPs. Necroptotic cells lose the integrity of their membrane, which leads to the release of their DAMPs, which include HMGBl and IL-1. **B** The GSDMD-N is released and translocated to the inner plasma membrane together with PS, which is exposed to the outer membrane as a ‘eat-me’ signal when the inflammasome is active. The oligomerisation induces transmembrane pores to open, the production of pro-inflammatory cytokines like IL-1 and IL-18, and cell swelling that triggers the release of DAMPs like ATP

Active caspase-1 not only cleaves and activates GSDMD, but it also activates the inflammatory cytokines IL-1 and IL-18 (Fig. 2). Caspase-4/5/11, which have been discovered to specifically engage cytosolic LPS, are what differentiate non-canonical inflammasomes from caspase-1 and result in GSDMD targeting and activation [52]. Caspase-4/5/11 does not directly process IL-1 and IL-18 [65], but their activity is sufficient to initiate the classical NLRP3 inflammasome and activate IL-1 by inducing GSDMD-mediated potassium efflux [66, 67]. Other membrane-damaging mechanisms, such as those driven by the mixed lineage kinase domain-like pseudo kinase (MLKL) or monosodium urate crystals, have been shown to trigger IL-1 production even in the absence of the GSDMD pore [68, 69].

Pyroptosis and necroptosis are both types of inflammatory lytic cell death. However, pyroptotic and necroptotic death may serve different functions, as evidenced by their distinct genetic drivers. Caspase 8 inhibition of fatal necroptotic signalling, for example emphasises that necroptosis is primarily recognised as a backup cell death defence system activated when apoptosis is inhibited [70, 71]. Pyroptosis, on the other hand, is a basic cellular response triggered by the identification of potentially harmful insults such as pathogen ligands, DAMPs, elevated prevalence of host metabolites and environmental irritants [72, 73].

GSDMD is activated by pyroptosis cleavage during proteolytic, whereas MLKL is activated by phosphorylation during necroptosis. MLKL’s equilibrium will shift from inactive cytoplasmic monomeric MLKL to membrane-associated MLKL oligomers as a result of RIPK3 phosphorylation (Fig. 1) [74–76]. Furthermore, the GSDMD-N domain has shown the monomer-to-oligomer transition, which a critical stage in MLKL’s ability to disrupt membranes [46, 58, 76]. Current studies on the physiological implications are focusing on whether GSDMD and MLKL-induced membrane destruction is sufficient to allow the production of DAMPs and other small soluble cytosolic components prior to complete cell lysis. However, evidence suggests that prelytic GSDMD holes may allow for ion flux or cytokine secretion even before to plasma membrane rupture [77]. Numerous studies have also suggested this possibility, including those that found MLKL/GSDMD-mediated or GSDMD-independent IL-1 secretion prior to cellular lysis or in the absence of cell death [68, 78–80].

MLKL and GSDMD killing can result in a variety of morphologies. Changes in MLKL and GSDMD targeting and pore-creation processes, as well as experimental variations in the kinetics of necroptotic and pyroptotic cell death, may aid in understanding. The non-selective diffusion of ions produced by GSDMD-N whole creation is thought to cause decreased cell enlargement and demolished cytoplasm during pyroptosis, and cells going through pyroptosis maintain adhesion until the plasma membrane is damaged [76, 77]. While disruption to the ion-selective MLKL protein affects intracellular osmolarity, causing cell swelling and osmolysis, necroptotic signalling causes cellular detachment [76]. Because all three types of cell death involve the breakdown of the plasma membrane, Phosphatidylserine Annexin V staining cannot distinguish between apoptosis, pyroptosis and necroptosis [77].

## Efferocytosis of necroptosis and pyroptosis

Efferocytosis typically terminates apoptosis by preventing the accumulation of dead cells, inflammatory reactions and secondary necrosis [81, 82]. Efferocytosis is the phagocytosis of dying and dead cells as well as their debris by phagocytes [83, 84]. Efferocytosis, which results in uptake into ‘spacious phagosomes’, combines characteristics of traditional phagocytosis and the liquid absorption process macro pinocytosis [85, 86]. Although efferocytosis distinguishes the identification and digestion of dead and dying cells from other types of phagocytosis [84, 87], its molecular properties are unknown. Efferocytosis necessitates the use of a large number of soluble and cell surface receptor-ligand interactions that have been identified for phagocytosis. Efferocytosis is controlled by several communications between the phagocyte and its dying target cell. Originally, efferocytosis was defined as the removal of apoptotic cells, but this definition has since been expanded to include other types of cell death [86].

Efferocytosis is carried out by professional and non-professional phagocytes, such as DCs, macrophages, fibroblasts and epithelial cells, by identifying find-me and eat-me signals from apoptotic cells [82]. Phagocytes interact with apoptotic cells via a set of signals known as ‘apoptotic cell-associated molecular patterns’, or ACAMPs [87]. Externalised phosphatidylserine, calreticulin and modified carbohydrates known as ACAMPs will be discussed briefly below. These compounds are recognised by a specific set of receptors and bridging molecules. Efferocytosis is compensated by four steps: (1) phagocyte recruitment controlled by find-me signals, (2) dead cell recognition controlled by eat-me signals, (3) dead cell absorption and (4) dying cell degradation [23, 88]. Healthy cells send tolerate I signals, also known as ‘keep-me’ or ‘do not eat-me’ signals, to prevent efferocytosis [15]. Some receptors, such as complement and antibody Fc receptors, communicate with the cytoskeleton and initiate direct phagocytic activity in response to eat-me signals, whereas others, such as the TIM-4 receptor, only anchor the target cell [89, 90]. To determine whether or not to ingest the target cell, a phagocyte will combine information from multiple receptors [89–91]. Cellular material is completely swallowed via cytoskeletal remodelling of the plasma membrane [92–95]. The ingested cell is often, but not always, cleared within a phagolysosome-type compartment after processing [96–98]. During the target cell identification phase, phagocytes may also examine the target’s chemical components to assess the danger it poses, as well as its physical characteristics, such as size, shape and topography [99–101]. This analysis determines three things: (i) the fate of the target cell within the phagocyte; (ii) whether the clearance process is immunologically silent, such as apoptotic cells efferocytosis; and (iii) whether engulfment occurs or is replaced by, for example, neutrophil NETosis, an anti-microbial cell killing process in which neutrophils expel chromatin extracellular snares [90, 102, 103].

### Find-me signals

Apoptotic cells emit find-me signals in order to distinguish themselves from healthy cells and to attract phagocytes to areas of death [15]. These signals primarily function as DAMPs, promoting the production of a variety of cytokines and chemokines that activate phagocytes [3, 4]. Sphingosine-1-phosphate (S1P), lysophosphatidylcholine (LPC), nucleotides and C-X-C motif chemokine ligand 1 (CX3CL1) are components of find-me signals [104–107]. Sphingosine kinases generate S1P from sphingosine, which regulates phagocyte cell migration by interacting with G-protein-coupled receptors ([104, 108], whereas caspase-3 and phospholipase A2 generate LPC [109]. Nucleotides such as uridine diphosphate (UDP) and adenosine triphosphate (ATP) promote phagocyte engagement with purinergic receptors, resulting in the phagocytic clearance of apoptotic cells [106]. Apoptotic cells secrete the chemokine CX3CL1 under the control of caspase and Bcl-2. By interacting with CX3CL1 and the macrophage fractalkine receptor (a find-me signal), macrophages are directed to apoptotic sites [105]. However, the molecular mechanisms underlying this process are still poorly understood. While all of these factors may cause macrophages to apoptotic cells, the significance of specific find-me signals in efferocytosis is dependent on a variety of factors, including phagocyte and apoptotic cell type, as well as the apoptotic impulses and phase of apoptotic cell death being studied (reviewed in [110]). Several of these find-me signals are also important regulators of macrophages inflammatory responses, as will be discussed further below.

Apoptotic cells can release adenine and uridine nucleotides from their surface via hexametric pannexin-1 channels activated by caspase-3/7 [107]. Necrosis, inflammatory cells and all caspase-dependent processes, including necroptosis and pyroptosis, also release these nucleotides (Fig. 2) to speed up cell removal, the extracellular nucleotides ATP and UTP act as ‘find-me’ signals [111]. They achieve this by increasing the number of P2Y purinergic-expressing motile phagocytes and upregulating phagocytic receptors [106, 112, 113].

Extracellular nucleotides can influence macrophage immune responses by converting ATP to adenosine, a well-known and powerful regulator of macrophage inflammation [111, 114]. Recent research has shown that during efferocytosis, Gs-linked A2a and A2b adenosine receptors on macrophages reduce pro-inflammatory cytokines (such as the C-X-C motif chemokine ligand 1 (CXCL1) and CXCL2) and increase pro-resolution factors (such as Nr4a, Thbs1) [115, 116]. The specific pathways involved in the production of extracellular adenosine during efferocytosis are unknown. Although adenosine can be transferred directly from macrophages (117), extracellular adenosine accumulates in a variety of tissue conditions as a result of ecto-enzymes like CD39 (ATP/ADPAMP) and CD73 (AMP adenosine) hydrolysing extracellular adenine nucleotides (ATP, ADP and AMP) [111, 114]. It is unknown how these ecto-enzymes influence adenosine synthesis and macrophage immunology during efferocytosis. According to Wang et al. research, pyroptotic cells use ATP as a find-me signal to attract macrophages. They also discovered that necroptotic cells caused THP-1 cells to migrate in a Trans well migration study. Additional research discovered that the activity of ATP released by necroptotic cells as a find-me signal caused THP-1 cell migration [117].

In contrast to the clearance of apoptotic and necrotic cells, the clearance of dead cells via alternate cell death pathways, such as necroptosis, is only now being reported. When caspase inhibition is present, as it is during viral infections, the RIPK1/3 and MLKL factors drive necroptosis and necroptosis can be induced via the TNF pathway. As a result, necroptosis does not typically involve the activation of several of the caspase 3/7-induced essential regulators (such as PANX1 and Xrk8) required for clearance systems (such as ATP production and PS presence) (Fig. 2) [118]. Recent research has shown that the critical necroptotic regulators RIPK3 and MLKL are required for necroptotic cells to reveal PS prior to membrane permeabilization [119, 120]. Thus, increased phagocytic receptor TIM4 levels can improve necroptotic cell removal, and the PS binding protein Milk fat globule (MFG) epidermal growth factor 8 (MFG-E8) can be used to identify necroptotic cells [120]. In addition to PS, the lipid mediator Resolvin D1 may aid in the clearance of necroptotic cells by promoting phagocytic CRT production, which identifies and makes it easier to identify necroptotic bone marrow-derived macrophages (BMDM) [121]. Necroptotic bodies are small PS-positive extracellular vehicles released by necroptotic cells that look like apoptotic bodies [119, 120], the question of whether these necroptotic bodies have any bearing on the efficacy of necroptotic cell removal remains unanswered.

The signalling pathways underlying the elimination of pyroptotic cells are currently being described, similar to the mechanism for necroptotic cell clearance. Similar to how PANX1 is cleaved by caspase 3/7 during apoptosis, PANX1 is triggered by caspase 1/11 during pyroptosis and helps generate ATP ‘find-me’ signals to promote phagocytic migration. In order to attract phagocytes, pyroptotic cells release IL-1 and IL-18 through GSDMD pores in a manner independent of cell lysis (Fig. 2) [60, 122]. In comparison to apoptotic ‘find-me’ signals, necroptosis and pyroptosis cells may lack the ability and power required to interpret their own signals. The majority of the molecules found to be produced by dying cells are DAMPs, which are actual biological elements that are normally hidden within the cell but become visible to the outer membrane when the cell injured or death. DAMPs include the nuclear protein high mobility group box-1 protein (HMGB1), N-formylated peptides derived from mitochondria, RNA, DNA, ATP, uric acid, actin, histones, calcium-binding S100 proteins and heat-shock proteins (Fig. 2) [123]. Additionally, necrotic cells mare lease accumulating inflammatory mediators such as IL-1, IL-33 and chemokines. These mediators may either directly or indirectly attract phagocytes to the area. Furthermore, when necrosis occurs, the complement and coagulation proteolytic cascades are immediately activated, exposing ‘unique’ molecules. The powerful chemoattractant C5a is one of the ‘find-me’ signals produced by complement activation on necrotic debris [124]. Even though necroptosis and pyroptosis are both parts of the necrotic process, the mediators that are released during each of them may be different from those that are released during necrosis as a whole. However, research into these systems is ongoing. A brief description of additional necrosis mediators is provided below.

In the scientific literature, formyl-peptides have a well-established role as a necrotic ‘find-me’ signal. In seminal studies using targeted thermal injury to the liver, formyl-peptide receptor 1 (FPR1) activation of neutrophils was the critical process required for mobility into the necrotic area [125]. Formyl-peptides bind to the FPR1, FPR2 and FPR3 receptors, but the classic chemotactic effects are primarily caused by FPR1 activity. An intravascular gradient of CXC chemokines aided in the initial migration of neutrophils toward the liver. Clinically relevant drug-induced liver damage and hepatic ischaemia–reperfusion disease models have revealed neutrophils’ reliance on formyl-peptide gradients for migration to necrotic areas [126], while the majority of research has focused on formyl-peptide-induced neutrophil chemotaxis and activation, macrophages also express FPR1 and respond to formyl-peptide stimulation. When mitochondrial extracts containing formyl-peptide are present, human peripheral blood mononuclear cells significantly release the CXCL8 [127], it is worth noting that when formyl-peptides are combined with other immunostimulatory DAMPs, such as HMGB1, the response is improves. This demonstrates that formyl-peptides can stimulate existing macrophages to produce more chemo attractants, which could then attract phagocytes to necrotic areas in an indirect manner (CXCL8).

Chemotactic cytokines, or chemokines, control where and how leukocyte populations are recruited within an organism. When there is necrosis, chemokines can be produced by both damaged and healthy observer cells, acting as both primary and secondary ‘find-me’ signals. Almost all cell types, including resident leukocytes, can produce CC and CXC chemokine’s [128], the chemokine CXCL1 can be released by endothelial cells, hepatocytes, macrophages, pericytes and fibroblasts. Kupffer cells, for example, express CCL2 after necrotic injury, whereas neutrophils release CXCL2 during transendothelial migration [129]. The fact that there are various chemokine sources that can trigger phagocyte migration to necrotic sites demonstrates the importance of chemokines as necrotic ‘find-me’ signals.

Leukotriene B4 (LTB4) is another find-me signal that increased cell movement in necrosis. LTB4 is a phospholipid facilitator that is synthesised from lipid membranes. LTB4 is a powerful neutrophil chemoattractant. When it comes into contract with GI, it stimulates the BLT1/LTB4R1 receptor, causing Rho GTPases and Src-family kinases to pair with it and accelerate neutrophil motility [130]. Neutrophils recruited to the region that produces LTB4 to boost other neutrophil recruitment to necrotic foci and form the recognisable densely populated clusters associated with neutrophil swarming. Neutrophil LTB4 can act as a signal relay molecule required for cell–cell interaction to increase neutrophil aggregation at the damaged site. LTB4 has also been found to collaborate with other necrotic ‘find-me’ signals, such as formyl-peptides and chemokines, implying that there is not just one necrotic ‘find-me’ signal, but rather a collaborative pool of signals with varying chemotactic potencies and ranges that work together to facilitate an effective response [131, 132].

Organelles such as mitochondria and phagosomes frequently produce reactive oxygen species (H_2_O_2_). Leukocytes must be equipped with a method for detecting the transitory H_2_O_2_ gradient produced by damaged cells. According to preliminary research, the redox sensor is the Src-family kinase Lyn, which is activated by wound-derived H_2_O_2_ and facilitates neutrophil migration to damaged areas in zebra fish [133]. H_2_O_2_ oxidises cysteine C466, activating Lyn and allowing neutrophils to migrate to the wound. Except for T cells (which express related Src-family kinases), all mammalian leukocytes express Lyn and human and murine neutrophils are also chemotactic to H_2_O_2_ [133, 134]. As a result, H_2_O_2_ acts as a necrotic ‘find-me’ signal for several types of leukocytes. In addition to its direct effects on phagocyte migration to injury sites, H_2_O_2_ can modify other ‘find-me’ signals such as fMLP, LTB4 and CXCL8 (141). Indeed, the NADPH oxidase at the leading edge of neutrophils is required for the generation of reactive oxygen species, which oxidise and inhibit the phosphoinositide phosphatase PTEN. This keeps PI (3, 4 and 5) P3 levels high at the leading edge and promotes neutrophil directed movement [135].

One of the first substances produced by injured and dead cells is nucleotides [136]. Nucleotide detection is mediated by the P2Y and P2X receptor families, which are G protein-coupled receptors and nucleotide-gated ion channels, respectively. Although there are several of these receptors, each with different sensitivity to different nucleotides (such as ATP, ADP and UTP), the majority of research has focused on the function of ATP and its breakdown products. Apoptotic cells initially used ATP as a ‘find-me’ signal [106]. the researchers discovered that P2Y2-dependent ATP and UTP produced during apoptosis are required for monocyte migration toward apoptotic cell supernatants. Furthermore, the absence of P2Y2 prevented monocytes from migrating toward apoptotic cells in vivo. Even more intriguing is the role of purinergic signalling in necrotic injuries. Using a focused necrotic lesion to the liver, it was demonstrated that ATP is required for peritoneal macrophage incursion into the necrotic site [137].

### Eat-me signals

Apoptotic cells attach to cell surface receptors such as stabilin-1 and stabilin-2, adhesion G protein-coupled receptor B1, T cell immunoglobulin mucin receptor (TIM) 1, TIM3 and TIM4 in the second stage of efferocytosis [138–142]. This would have pleiotropic effects via a number of bridging molecules, such as protein S and MFG epiderma, this would have pleiotropic effects [143–145]. Similarly, transglutaminase 2 (TG2) binds to MFG-E8 and acts as an activator of Rac 1 by acting as an integrin 3 receptor. As a result, apoptotic cells are digested. Integrin 3, on the other hand, is unable to recognise apoptotic cells in the absence of TG2 [146]. Phosphatidylserine (PS), which is found in the inner membrane of cells and is produced externally by caspase signals upon death, appears to have a significant impact on eat-me signals [147, 148].

Eat-me signals are either directly recognised by PS binding receptors or indirectly recognised by phagocytes bonding facilitators. PS is abundant on the exofacial side of the membrane, where it is normally restricted to the inner leaflet of live cells, as a result of caspase-mediated changes in the activity of numerous important phospholipid transport enzymes. There are currently at least 12 PS efferocytosis receptors identified, which are a group of surface proteins with a variety of structural properties that can either directly or indirectly bind to PS by recognising soluble PS binding opsonins [149]. The receptor for advanced glycation end products (RAGE) can recognise PS and is involved in the efferocytosis in macrophage [84]. MFG-E8 recognises PS and is recognised by the αVβ3 and αVβ5 phagocyte receptors (such as those on DCs and macrophages). When these receptors engage, the cytoskeleton may change, promoting the absorption of apoptotic cells [150–152]. Furthermore, soluble CD93 interacting with PS and integrin × 2 on apoptotic cells induces efferocytosis via an opsonin, and endothelial and phagocytic cells detect the interaction of the complement factor C1q with PS [153]. These findings suggest that being exposed to PS may increase the engulfment of dying cells. PS is recognised by membrane number receptors such as Stabilin-1, Stabilin-2, TIM4, RAGE, BAI-1 and CD300. PS receptors have been shown to be required for identifying dead cells [139, 140, 142, 154]. Macrophages that express stabilins 1 and 2, for example, can detect PS on apoptotic cells and absorb more apoptotic debris [147, 155]. This method is necessary for identifying and removing PS-stimulated aged or injured erythrocytes. During apoptosis CD300 can detect PS and phosphatidylethanolamine [156]. As a result, a lack of CD300f and CD300d can impair normal macrophage efferocytosis [157]. PS is detected by scavenger receptors (SR) SR-A1, SR-B1 and CD36, which stimulates macrophage efferocytosis [156]. It is well known that the traditional DAMP, HMGB1, inhibits RAGE/PS-mediated efferocytosis in macrophages by binding to integrin v3 [158]. While HMGB1-deficient macrophages effectively phagocytize apoptotic neutrophils and thymocytes [159], HMGB1 is translocated into the cytoplasm before being released into the extracellular environment [160]. Rac, CDC42, Rab5, Rho A and Rho-associated coiled-coil kinases (ROCK) members of the Ras homolog family (Rho) of small GTPases are also important in regulating the absorption of dying cells [161–163].

Overall, the literature shows that apoptotic cell clearance dominates the topic cell elimination [164–166]. Phagocytic receptors may be unable to recognise necrotic cells because they contain varying amounts of PS [164]. As a result, necrotic cells may take longer to be digested by phagocytes than apoptotic cells [165]. Furthermore, necrotic cells frequently produce a single massive bleb and continue to exist as a single biological entity, as opposed to apoptotic cells, which rapidly bleb and divide into apoptotic bodies [166]. Given the importance of dying cell breakup in facilitating cell elimination, this could also explain why necrotic cell removal is less efficient than apoptotic cell absorption and the many mechanisms involved [165, 167]. It has been demonstrated in both in vitro and in vivo experiments clearing apoptotic cells is more effective than engulfing necroptotic or pyroptotic cells, as well as clearing necroptotic cells [164, 167, 168]. However, contradictory results have been observed [166].

Exposed PS can also be found in necroptotic cells after phosphorylated mixed lineage kinase-like (pMLKL) translocation to the membrane (Fig. 2). Necroptotic cells exposed to PS produce extracellular vesicles transporting pMLKL and proteins. Furthermore, after exposure to PS, pMLKL suppression can prevent necroptosis and restore cells. Finally, PS externalisation by necroptotic cells promotes phagocytosis and recognition, which may help to reduce the inflammatory response to this nonapoptotic form of cell death. Because of the specific find-me and eat-me signals produced when PS is exposed to the outer membrane and extracellular vesicles, necroptotic cell death may provide an immunologically silent window [167].

Similarly to necroptotic cells, pyroptotic cells emitted a find-me signal that was inhibited by apyrase, which converts nucleoside triphosphate to nucleoside monophosphate. According to Wang et al., findings pyroptotic cells caused by microbial infection can be effectively engulfed by either mice peritoneal macrophages or human monocytic THP-1-cell-derived macrophages. This engulfment was inhibited by the D89E mutant of MFG-E8, a phosphatidylserine-binding protein that has previously been shown to disrupt phosphatidylserine-dependent engulfment of apoptotic cells by macrophages. They found that after being treated with muramyl dipeptide, both pyroptotic and apoptotic cells adhered to a T cell immunoglobulin and mucin domain-containing 4 (Tim4; an additional phosphatidylserine-binding protein), whereas necrotic cells that had been destroyed by heat did not. This demonstrated that phosphatidylserine was activated in pyroptosis and apoptosis but not in necrosis [164]. When phagocytes are drawn to the area of cell death, they will interact with PS that is has appeared on the pyroptotic cell outer membrane either directly through scavenger receptors (TIM4) or indirectly through bridge molecules (MFG-E8). Because the mechanism is caspase 1 independent, it is unclear whether PS exposure occurs actively or passively during pyroptosis [58]. Given that the phospholipid scramblase TMEM16F can be activated by Ca2+ signalling, it would be interesting to see if such scramblases result in PS exposure during cell death mechanisms that do not involve caspase 3/7 activation, such as pyroptosis [118]. Apoptotic cells were the easiest for macrophages to consume, followed by pyroptotic cells and finally necrotic cells killed by heat. These findings suggest that pyroptotic cells, like apoptotic cells, actively stimulate macrophage phagocytosis by emitting eat-me and find-me signals [164].

Engulfment of dead cells is required for tissue homeostasis and the suppression of inflammatory responses. The process of engulfing apoptotic cells has been extensively studied, and it involves receptors on the engulfing cells that detect eat-me signals on the apoptotic cells’ outer membrane [82]. To maintain tissue homeostasis, necrotic cells must be eliminated because they may leak intracellular components that contribute to inflammation [167], as previously stated, in both in vitro and in vivo conditions, apoptotic cell clearance has been shown to be more efficient than necroptotic and pyroptotic cell engulfment [164, 167, 168]. Non-professional phagocytes digested necroptotic and pyroptotic cells much more efficiently than apoptotic cells in a study using NIH3T3 cells by Lu et al. Furthermore, they compared the ability of peritoneal macrophages, BMDM and bone marrow-derived dendritic cells to phagocytise apoptotic cells to necrotic and pyroptotic cells [164].

The processes of pyroptosis and efferocytosis in necroptosis are detailed in the previous chapter in order to reduce the pro-inflammatory effects of intracellular components that contribute to inflammation. Extensive research has been conducted on the cellular mechanism used to describe engulfed apoptotic cells. However, recent research has not revealed the cellular mechanism of necroptotic and pyroptotic cells after engulfment. As illustrated below, we believe that several cellular mechanisms in necroptosis and pyroptosis are similar to post-engulfment in apoptosis. More research is needed in necroptosis and pyroptosis to determine this.

After being recognised, classical apoptotic cells are absorbed by the efferocyte into an efferosome, a fluid-filled membrane vesicle. Efferosomes, like phagosomes that transport ingested infections, merge with early endosomes, late endosomes and then lysosomes in a highly controlled process [11]. Some of the proteins that control these merger processes are Rab GTPases and SNAREs and the merger activities deliver the hydrolytic enzymes that destroy the apoptotic cell inside the efferosome [169]. This is referred to as efferosome maturation, and it is similar to the maturation mechanisms observed following phagocytosis and endocytosis [97].

The activation of the Rab GTPases Rab5 and Rab7 is one of several similarities between the phagosome and efferosome maturation processes [139, 170]. Rab5 is attracted to efferosomes as the apoptotic cell is absorbed and remains firmly attached to them for a few moments after the cell membrane is exposed [171]. In this case, Rab5 promotes the fusion of the efferosome and the early endosomes, thereby initiating the catabolic process that eventually destroys the apoptotic cell [104]. Rab5 is converted to Rab7 shortly after the efferosome forms, and Rab7 mediates the merging of late endosomes and lysosomes to the efferosome to create a highly enzymatic hydrolysis environment capable of completely destroying the apoptotic cell [171, 172]. In contrast to phagocytosis, this efferosome maturation pathway also includes Rab17, which transports the efferosome’s broken contents to the renewing endosome, where they are exocytose. This inhibits their transfer to antigen loading compartments [172].

Furthermore, efferosomes have been shown to participate in LC3-associated phagocytosis (LAP), a process in which autophagy mediators, such as the class III phosphatidylinositol-3-kinase (PI3KCIII) complex ATG5 and ATG7, conjugate LC3 to the surface of developing efferosomes [173]. These variables then control the efferosome’s rapid expansion and the clearance of its apoptotic contents, preventing antigen presentation and polarising macrophages toward an anti-inflammatory phenotype [174]. The Medzhitov group demonstrated that antigen presentation requires contents to be directed into the conventional phagocytic pathway (for example, non-LAP). This pathway is dependent on phagosome-derived TLR signalling [138, 175]. This demonstrates that, in addition to inducing the activation of genes involved in inflammation and antigen presentation, TLR identification of pathogen products results in rapid changes in the trafficking of cargo containing TLR ligands versus cargo lacking these ligands [176]. Rab39a, GTPase that reduces autophagy in response to TLR stimulation and is required for the transfer of MHC I to phagosomes for antigen cross-presentation, may inhibit LAP after phagocytosis [177]. However, no studies on Rab39a’s function in efferocytosis have been published, so its role in efferocytosis-associated LAP is unknown.

Restricting the antigen presentation of efferosome-derived antigens is a critical reaction of phagocytes following efferocytosis, as evidenced by the existence of three concurrent mechanisms. These mechanisms include LAP, faster maturation and cargo redirected out of the maturing efferosome via Rab17 action. Efferocytes not only use non-trafficking processes, but they also prevent autoimmune reactions to efferocytosis materials. Efferocytosis is typically associated with increased levels of cytokines such as IL-10, which reduce the activity of mature T cells and promote the development of Treg cells from naive T cells [55]. As a result, T cell responses are suppressed by efferocytosis. It is critical to investigate this complex mechanism that engulfs necroptotic and pyroptotic cells. Small changes could have a different effect that benefits the drug.

## Conclusion

Cell death and the clearance of dead cells are linked to a number of inflammatory disorders. To develop new disease therapeutics, it is critical to understand the molecular mechanisms underlying phagocytic clearance and the functional implications of phagocyte engulfment. The inclusion of necroptotic and pyroptotic cells as a significant, continuous contributor to overall cell death provides a novel perspective on how debris are identified, eliminated and how it contributes to inflammation. The three steps of recruitment, recognition and engulfment contribute to the efficient efferocytosis in the necroptosis and pyroptosis pathways. These three phases are mediated by the exposure and release of the find-me, eat-me and engulfment signals. As previously stated, significant progress has recently been made in our understanding of the molecular mechanisms underlying pyroptosis and necroptosis. Several markers have been discovered, and some of them point to label linkages between various cell death pathways. According to current research, necroptotic and pyroptotic dying cells release several DAMPs as a find-me signal. PS is one of the most powerful eat-me signals recognised by phagocytes in necroptotic and pyroptotic cells. Despite significant advances in our understanding of efferocytosis in necroptotic and pyroptotic cells over the last few decades, there are still many unanswered questions. Concerns about the role of efferocytosis in pathogens elimination and efferocytes metabolic remodelling are two examples. Further investigation is required to comprehend efferocytosis in necroptosis and pyroptosis in order to understand the molecular mechanisms that contribute to the discovery of new markers and how they can be therapeutically targeted.

## Data Availability

Not applicable.

## Abbreviations

TNF: Tumor necrosis factor
FADD: FAS associated via death domain
DAMP: Damage-associated molecular pattern
RIPK: Receptor-interacting protein kinase
MLKL: Mixed lineage kinase domain-like protein
TLR: Toll-like receptor
PAMP: Pathogen-associated molecular patterns
DR: Death receptor
GSDMD: Gasdermin D
S1P: Sphingosine-1-phosphate
LPC: Lysophosphatidylcholine
CXCL: Chemokine (C-X-C motif) ligand
ATP: Adenosine triphosphate
HMGB1: High mobility group box-1
MFG: Milk fat globule
MFG-E8: Milk fat globule epidermal growth factor 8
BMDM: Bone marrow-derived macrophages
FPR: Formyl-peptide receptor
LTB4: Leukotriene B4
TIM: T cell immunoglobulin mucin
TG2: Transglutaminase 2
PS: Phosphatidylserine
RAGE: Receptor for advanced glycation end products
PMLKL: Phosphorylated mixed lineage kinase domain-like protein
LAP: LC3-associated phagocytosis
